# Healthcare provider perceptions on the implementation of the universal test-and-treat policy in South Africa: a qualitative inquiry

**DOI:** 10.1186/s12913-023-09281-2

**Published:** 2023-03-28

**Authors:** Edward Nicol, Vuyelwa Mehlomakulu, Ngcwalisa Amanda Jama, Mbuzeleni Hlongwa, Wisdom Basera, Desiree Pass, Debbie Bradshaw

**Affiliations:** 1 Burden of Disease Research Unit, South African Medical Research Council, P.O. Box 19070, Tygerberg, 7505 South Africa; 2 Division of Health Systems and Public Health, Faculty of Medicine and Health Sciences, Stellenbosch University, Stellenbosch, South Africa; 3 School of Public Health, University of the Western Cape, Cape Town, South Africa; 4 School of Nursing and Public Health, University of KwaZulu-Natal, Durban, South Africa; 5 School of Public Health and Family Medicine, University of Cape Town, Cape Town, South Africa

**Keywords:** Healthcare provider, HIV, PLHIV, Same-day ART, Universal-test-and-treat

## Abstract

**Background:**

South Africa had an estimated 7.5 million people living with HIV (PLHIV), accounting for approximately 20% of the 38.4 million PLHIV globally in 2021. In 2015, the World Health Organization recommended the universal test and treat (UTT) intervention which was implemented in South Africa in September 2016. Evidence shows that UTT implementation faces challenges in terms of human resources capacity or infrastructure. We aim to explore healthcare providers (HCPs)’ perspectives on the implementation of the UTT strategy in uThukela District Municipality in KwaZulu-Natal province.

**Methods:**

A qualitative study was conducted with one hundred and sixty-one (161) healthcare providers (HCPs) within 18 healthcare facilities in three subdistricts, comprising of Managers, Nurses, and Lay workers. HCPs were interviewed using an open ended-survey questions to explore their perceptions providing HIV care under the UTT strategy. All interviews were thematically analysed using both inductive and deductive approaches.

**Results:**

Of the 161 participants (142 female and 19 male), 158 (98%) worked at the facility level, of which 82 (51%) were nurses, and 20 (12.5%) were managers (facility managers and PHC manager/supervisors). Despite a general acceptance of the UTT policy implementation, HCPs expressed challenges such as increased patient defaulter rates, increased work overload, caused by the increased number of service users, and physiological and psychological impacts. The surge in the workload under conditions of inadequate systems’ capacity and human resources, gave rise to a greater burden on HCPs in this study. However, increased life expectancy, good quality of life, and immediate treatment initiation were identified as perceived positive outcomes of UTT on service users. Perceived influence of UTT on the health system included, increased number of patients initiated, decreased burden on the system, meeting the 90-90-90 targets, and financial aspects.

**Conclusion:**

Health system strengthening such as providing more systems’ capacity for expected increase in workload, proper training and retraining of HCPs with new policies in the management of patient readiness for lifelong ART journey, and ensuring availability of medicines, may reduce strain on HCPs, thus improving the delivery of the comprehensive UTT services to PLHIV.

## Introduction

Globally there were 38.4 million people living with HIV (PLHIV) at the end of 2021 and 28.7 million were initiated on treatment by the end of December 2021. [1, 2]. South Africa continues to have the largest number of PLHIV with an estimated 7.5 million, accounting for approximately 20% of the 38.4 million PLHIV in the world in 2021 [3, 4]. South Africa has achieved great strides in its response strategies on HIV treatment with the roll-out of ART that has led to a decline in HIV-related mortality rates [5, 6]. South Africa has been reported as having the largest HIV treatment programme in the world [7, 8]. The government has implemented several strategies to curb the disease, including the execution of the comprehensive ART Clinical Guidelines for the Management of HIV in Adults, Pregnancy, Adolescents, Children, Infants and Neonates [9].

In May 2016, following recommendations of the World Health Organization (WHO) [10], the South African government announced the progressive rollout of the universal test and treat (UTT), with an intention to reduce deaths and new infections by 2030 [11, 12]. The 2030 targets included the 95-95-95 targets, which state that 95% of HIV positive people should know their status, 95% of those who know their status should be on treatment and 95% of those on treatment should be virally supressed [13]. UTT advocates that all individuals testing for HIV be initiated on treatment regardless of their clinical staging or CD4 count [14, 15], which is a promising strategy to improve the health of PLHIV [16–19]. It aims to reduce the incidence of HIV infection through increased HIV testing and provision of immediate access to treatment.

Given the high HIV prevalence in South Africa, the reported benefits of early treatment initiation for PLHIV gives hope of reducing HIV incidence rates. Four large population-based randomized trials of UTT, the population effects of ART to reduce HIV transmission (PopART) [20], Botswana Combination Prevention Project (BCPP) [21], the sustainable East Africa research in community health (SEARCH) [22] and TasP [18, 19], have shown to some degree the benefit of UTT for reducing HIV transmission. For instance, the PopART trial conducted in South Africa and Zambia, which examined the impact of a package of HIV prevention interventions on community-level HIV incidence, showed a significant reduction in new HIV infections [20]. However, not all these trials have shown impact on reducing HIV incidence.

Despite the importance of UTT, evidence shows that the implementation of UTT in South Africa faces challenges such as increased non-retention rates [23–25], increased workload [24], leading to inadequate human resource [18, 26], and challenges with infrastructure [26, 27], human resources capacity and infrastructure [18, 24, 26, 28, 29]. Other potential barriers include psychosocial and health systems factors [27, 30], such as shortages of drugs [31, 32], long patient waiting times [33], leadership and governance [29], competing clinical care priorities [34], communication breakdown between HCPs and patients [35], insufficient skill and inadequate supervisory support [29]. These could have negative impacts on the effective implementation of UTT.

Healthcare providers (HCPs) are key players in the implementation of UTT in the health facilities [36, 37]. They are the ambassadors of the national Department of Health (NDoH) in advocating for the new HIV treatment guidelines particularly in terms of communicating new guidelines to those diagnosed with HIV. To identify the health system gaps, it is therefore important to understand how HCPs navigate the new changes and impacts to their health service delivery. In this study, we aimed to explore HCPs’ perspectives on the implementation of UTT in the uThukela district, KwaZulu-Natal province.

## Materials and methods

### Study design and setting

A qualitative study design approach was followed to understand the perceptions and experiences of HCPs on the implementation of the UTT strategy. The study was conducted in the uThukela District Municipality (DM) in KwaZulu-Natal (KZN), South Africa. uThukela DM is a predominantly rural district with high HIV prevalence of 22.4% [38] and shares its western border with the country of Lesotho. The district is comprised of three local municipalities (LMs) namely Alfred Duma LM, Inkosi Langalibalele LM and Okhahlamba LM.

Universal Test and Treat in the uThukela DM is implemented and delivered through multiple modalities of differentiated treatment delivery models. However, in the 18 facilities selected for this study (Table 1), UTT is delivered mainly through the facility-based and community-based models, which are integrated with other services such as those for patients with non-communicable diseases [39]. The facility-based model consists of HCP-led group of patients who meet regularly at the facility to receive health education, health screenings, adherence support, peer support, and collect ART; while the community outreach model, which are also nurse-led, provide both clinical care and medications at offsite areas in the community as an extension of the clinic (mobile clinics). In addition to the support provided by HIV counsellors, the use of case managers, linkage officers, and nurse clinicians have become critical in providing support to individuals diagnosed with HIV, in so far as complementing the initial counselling services provided by counsellors. These differentiated service delivery models have the potential to increase retention in care and adherence to medication among people living with HIV [40].

**Table 1:** Selected facilities included in the *Linkage* to Care study in uThukela district between December 2017- August 2019

	Local Municipality	Name of facility	Type of facility/service
**1.**	Alfred Duma	Ladysmith Gateway Clinic	Gateway Clinic
**2.**		Limehill Clinic	Clinic
**3.**		Outer West Mobile 1	Mobile
**4.**		Sigweje Clinic	Clinic
**5.**		St Chads CHC	CHC
**6.**		Walton Clinic	Clinic
**7.**		Steadville Clinic*	Clinic
**8.**		Watersmeet Clinic*	Clinic
**9.**	Okhahlamba	Bergville Clinic	Clinic
**10.**		Bergville Mobile 2	Mobile
**11.**		Bergville Mobile 3	Mobile
**12.**		Emmaus Gateway Clinic	Gateway Clinic
**13.**		Emmaus Hospital*	OPD
**14.**	Inkosi Langalibalele	Connor Street Clinic	Clinic
**15.**		Estcourt Gateway Clinic	Gateway Clinic
**16.**		Injisuthi Clinic	Clinic
**17.**		Ntabamhlophe Clinic	Clinic
**18.**		Wembezi Clinic	Clinic

*Facilities that were not part of the original sample but added later to replace facilities with low enrolment rates

**Community Health center (CHC**): A facility that normally provides PHC services, 24-hour maternity, accident and emergency services and beds where health care users can be observed for a maximum of 48 hours, and which normally has a procedure room but not an operating theatre. **Clinic**: A facility at and from which a range of primary health care services is provided and that is normally open eight or more hours a day based on the need of the community to be served., **Gateway clinic**: A primary health care (PHC) clinic, located in community health center (CHC) or a hospital Out-patient department (OPD) where patients with minor ailments are seen by trained primary health care workers free of charge, before being referred to the CHC or hospital. Every CHC/ hospital has a gateway clinic directly attached to it to serve the people in the immediate vicinity of the CHC.

### Conceptual framework

To analyse HCPs’ experience and perceptions providing HIV care under the UTT strategy, an adapted version of the framework for a systems approach to healthcare delivery (FSAHD) [41] was used. This four-level model of the health care system consists of (a) individual patient (the service users); (b) the care team or frontline workers, i.e., HCPs, patients’ family members and other care givers; (c) the organization that provides the infrastructure and resources for care such as hospital, clinic, and nursing home. The fourth level in this model is the political and economic environment such as the public and private regulator, insurers, healthcare purchasers, research funders that influence the structure and performance of health care. For analysis purposes, this study will only focus on the first three levels.

### Key informant interview procedures

A sample of 161 HCPs were purposively selected from 18 health facilities and three sub-districts, based on their experience implementing the UTT programme (Table 1). These consisted of at least two HPCs from each available cadres comprising of Managers (facility managers and PHC manager/supervisors); Nurses (enrolled, auxiliary, and professional Nurses) and Lay workers (lay counsellors, community health workers, linkage officers).

### Data collection procedure

An open-ended questionnaire adapted from Hoffman et al., 2015 [42] was used to collect qualitative data from HCPs. This structured interview included data on demographic characteristics, work experience and HCPs’ perceptions on the implementation of UTT. Participants were approached by trained interviewers and depending on availability, an appointment was secured in order not to disrupt the daily functioning of the health facilities. In most instances the interview, which lasted between 20 and 105 minutes, was conducted over more than one visit depending on the availability and time constraints of the individual. The interviews were audio recorded, and simultaneously captured into REDCap by the interviewers to reduce the time spent on transcriptions.

Each respondent had a unique code, e.g., BISC-EN-M, which are added at the end of each quote to provide context. The first initial letters describe the facility area and the type (i.e., primary clinic, mobile clinic etc.), followed by participants’ job category (e.g., enrolled nurse - EN) and gender (F/M) at the time of the interview.

### Data management and analysis

The recorded interviews were transcribed verbatim and updated in REDCap and exported to Excel. The analysis team, which comprised of three members (EN, VM, NAJ), independently read through all the transcripts to gain a general understanding of the content and scope, and discussed initial emerging themes used to develop a structured coding framework. Coded information was linked to broader emerging themes across interviews. Data from HCP interviews were coded independently using ATLAS.ti v8 software. Comparative analysis between two skilled qualitative researchers was performed to ensure analysis accuracy. The analysis process was informed by the interview questions, literature, and the inductive approach to the data.

## Results

Of the 161 HCPs included in this study, 41% (66) were from Alfred Duma local municipality where eight of the 18 health facilities are located (Table 2). The study consisted of 97.5% (n = 157) Black African participants, of which 88.2% (142) were female, and most 90% (145) had obtained a tertiary level of education. While a fifth of the participants (33) were professional nurses, most participants (98.1%, 158) were employed in a public clinic setting (Table 2).

**Table 2:** Demographic characteristics of HCPs in the Linkage to Care study disaggregated by subdistrict in uThukela district − 2019

Characteristics	Total (n = 161)	Alfred Duma (n = 66)	uKhahlamba (n = 49)	Inkosi Langalibalele (n = 46)
**Job category, n (%)**				
Facility manager	17 (10.6)	8 (12.2)	6 (12.2)	3 (6.5)
Professional nurses	33 (20.5)	13 (19.7)	10 (20.4)	10 (21.7)
Lay counsellors	30 (18.6)	11 (16.7)	10 (20.4)	9 (19.5)
Community health workers	19 (11.8)	6 (9.1)	6 (12.2)	7 (15.2)
PHC Manager/ Supervisor	3 (1.9)	1 (1.5)	1 (2.1)	1 (2.2)
Linkage officers	10 (6.2)	5 (5.6)	1 (2.1)	4 (8.7)
Enrolled nurses	34 (21.1)	15 (22.7)	10 (20.4)	9 (19.5)
Enrolled nursing assistants/ Auxiliary	15 (9.3)	7 (10.6)	5 (10.2)	3 (6.5)
**Level of work, n (%)**				
Facility	158 (98.1)	65 (98.5)	48 (98.0)	45 (97.8)
Sub-district	3 (1.9)	1 (1.5)	1 (2.0)	1 (2.2)
**Sex, n (%)**				
Male	19 (11.8)	11 (16.7)	6 (12.2)	2 (4.4)
Female	142 (88.2)	55 (83.3)	43 (87.8)	44 (95.7)
**Ethnicity, n (%)**				
Black African	157 (97.5)	64 (97.0)	49 (100)	44 (95.7)
Coloured	2 (1.2)	1 (1.5)	0	1 (2.2)
Indian/Asian	2 (1.2)	1 (1.5)	0	1 (2.2)
**Education level, n (%)**				
High School	4 (2.5)	0	3 (6.1)	1 (2.2)
Matric	12 (7.5)	3 (4.6)	4 (8.2)	5 (10.9)
Certificate	64 (39.8)	26 (39.4)	19 (38.8)	19 (41.3)
Diploma	50 (31.1)	26 (39.4)	12 (24.5)	12 (26.1)
Post graduate diploma	8 (5.0)	1 (1.5)	5 (10.2)	2 (4.4)
Degree	14 (8.7)	6 (9.1)	5 (10.2)	3 (6.5)
Post graduate degree	9 (5.6)	4 (6.1)	1 (2.0)	4 (8.8)

### Perceptions on the implementation of UTT

**Fig. 1:**
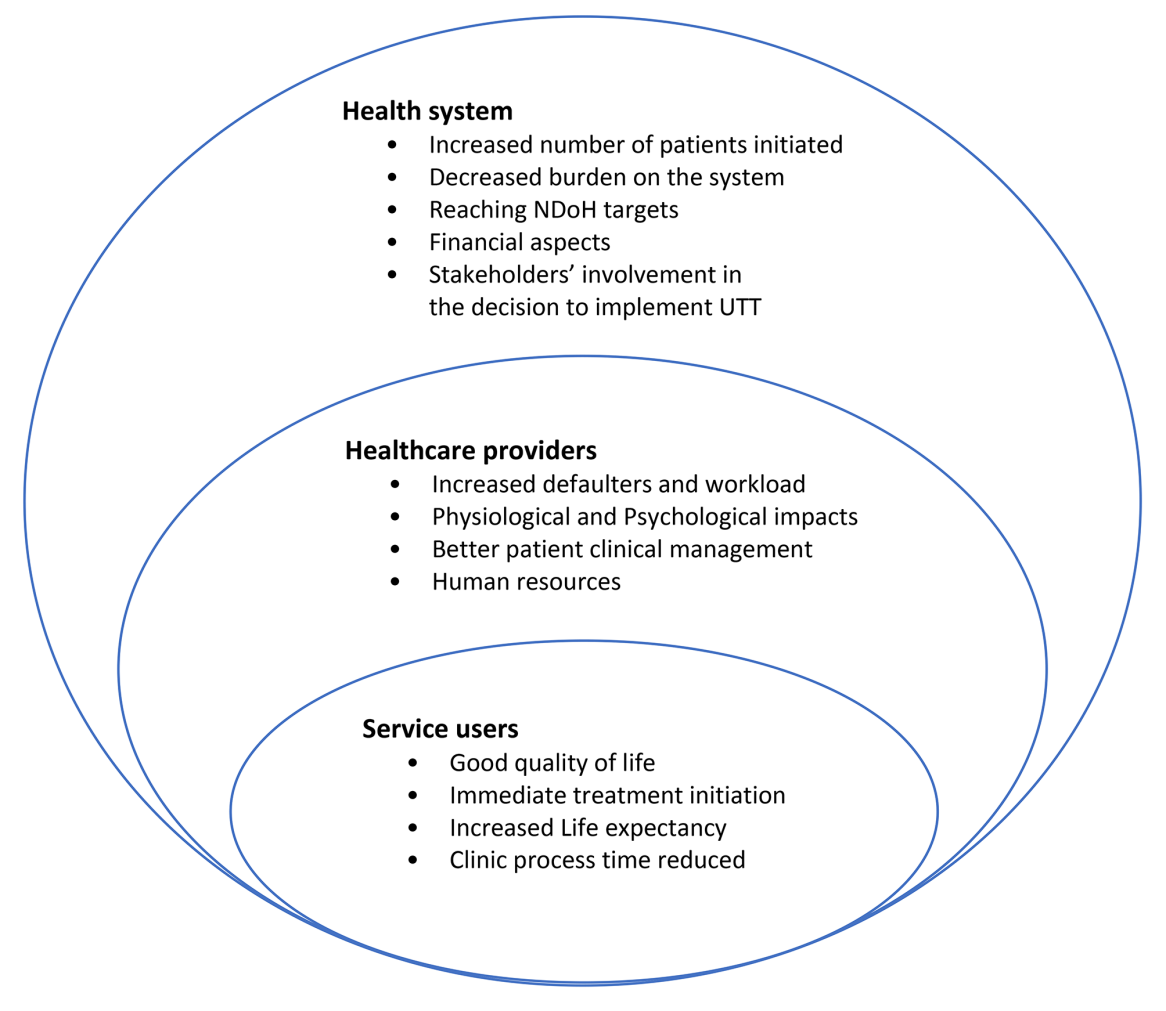
Themes relating to HCPs’ perceptions on the implementation of UTT by levels

Several themes relating to HCPs’ perceptions on the implementation of UTT were identified and classified under three levels according to the adapted framework for a systems approach to healthcare delivery (FSAHD) [41] i.e., health service users (patients), healthcare providers and health system (Fig. 1). Thirteen themes emerged from the data and are presented in detail with illustrative quotes.

### Perception of UTT on service users

Almost all interviewed HCPs reported positive effects of UTT on service users, such as helping the patients to live longer and healthier. These were some of the themes that emerged:

#### Good quality of life and immediate initiation in care

The general perception among HCPs was that UTT provided people living with HIV (PLHIV) the opportunity to have a good quality of life through immediate access to prevention and care services, which potentially increases early retention in care and adherence to medication.

*“It [UTT] has given them [PLHIV] the opportunity to easy access to treatment and services. It has prevented unnecessary sickness.” ****(BNHC005_PN_F)***

*“It [UTT] is a good impact, people are getting primary prevention, they are getting treatment early, which prevents other diseases from manifesting.” ****(KEMG012_PN_F)***

*“It [UTT] doesn’t get to a stage where someone is very sick, early treatment is helping them out.”* ***(KEMG002_EN_F)***

#### Increased life expectancy

Most of the participants agree that the implementation of UTT has led to increased life expectancy among PLHIV, with more people living a healthy life.

*“Good impact because they now live longer, and the stigma percentage have dropped.” ****(KEMP008_EN_F)***

*“It [UTT] has a very positive impact because they start treatment while the CD4 count is still alright, which reduces the number of infections; people live longer and are protected.”* ***(DIGC002_LC_F)***

*“It [UTT] has increased life expectancy and more patients are living a healthy life.”* ***(DIGC005_PN_F)***

#### Reduced clinic process time

Narratives also suggest that the time spent by patients accessing care in the clinics has reduced and that clinics are not as congested as were the case pre-UTT. Participants attributed the reduced waiting time and reduced congestion at clinics to the implementation of the UTT strategy.

*“It [UTT] has reduced the waiting period of patients at the clinic, because people are being quickly treated which reduces the number of people coming to the clinic.” ****(BNHC011_EN_F)***

*“It [UTT] has also made things easy for patients, because they come once a month to fetch treatment, after they have been diagnosed with HIV and also everything (initiating, HIV classes and taking bloods) is done on the same day, patient doesn’t have to keep on coming back to do all these things on a weekly basis.”* ***(KB2M002_LC_F)***

### Perception of UTT on healthcare providers

HCPs expressed different views on the effects the new UTT intervention has on them. While majority of participants reported on the negative effects of UTT on HCPs, there were some positive effects. These include increased workload, increased defaulters, physiological and psychological impacts, improved processes at the facilities, better patient management, and human resources.

#### Increased defaulters and workload

One of the major challenges reported by the HCPs was that UTT implementation increased patient defaulter rates due to patients’ unreadiness to initiate ART. HCPs expressed that UTT creates a high number of ART defaulters, which is believed to have been caused by overwhelming feeling of starting a lifelong treatment immediately after diagnosis. According to the participants, some patients are not ready to start treatment since they are yet to accept their new HIV status, and as such would like some time to process the new reality of living with HIV. However, because they respect health care workers, they go along with the same day initiation post HIV positive diagnosis and some never get retained in care.

*“I feel like it [UTT] creates a lot of defaulters, because most people who test positive before they accept their status, ...are given treatment which they take just to please you as a health care worker, while they are not ready to take treatment; so, they just take it once off and never come back.”* ***(DLHC001_CM_F)***

*“…It [UTT] also have some disadvantages which is that we initiate them [HIV patients] same day assuming they are ready to start, yet they have not accepted [the new status] and they never come back and sometimes they provide incorrect information and when tracing them we never find them.”* ***(ECHC008_PN_F)***

Healthcare providers reported that the increased volume of defaulters is one of the reasons for increased workload causing a greater burden on them. The other reason for the increased workload reported by the HCPs was that UTT process is long, and counsellors have to provide all necessary services to patients in one session.

*“More work as counsellors have to do everything in one session with clients.”* ***(BISC008_CHW_F)***

*“More work for them as they have to account also for patients, why they did not start ART.”* ***(BISC006_CHW_F)***

*“The level of work has increased because they [HCPs] have to do post counselling, initiating, doing baseline bloods.”* ***(DIGC006_NA_F)***

However, other HCPs saw the increase in workload as a good opportunity to improve the processes in the facilities. They stated that:

*“There is more paperwork, but the good side is that health care workers get a chance to do everything with the client in one session.”* ***(BISC005_PN_F)***

*“It [UTT] has created more work which has a good impact because that has cut out unnecessary delays.”* ***(BNHC003_LC_F)***

#### Physiological and psychological impacts

Another negative side of UTT is the effect it has on the well-being of HCPs, which include some physiological and psychological impacts such as stress and burnout. One clinic manager and professional nurse highlighted issues of burnout and being overworked. Others highlighted issues around feeling under pressure and stressed.

*“Burnout on staff” ****(BISC004_CM_F)***

*“It [UTT] has created a lot of stress and demand due to targets that has to be met.”* ***(BNHC005_PN_F)***

*“UTT has made working easy but there is so much pressure ….”* ***(BISC009_EN_F)***

#### Better patient clinical management

Some clinic managers expressed several benefits of UTT on patient clinical management, while others commented on the advantage of UTT as it has improved the clinic flow and operations. HCPs felt that the introduction of UTT has improved patient management and care compared to the old HIV guidelines. Mostly because they do not have to treat very sick patients since UTT promotes same-day initiation after diagnosis.

*“[UTT] has many benefits and avoids clients coming constantly to facility and decreases mortality rate. Clients also feel welcome in clinic because they immediately receive medication.” ****(ELSG015_CM_F)***

*“It [UTT] is good because before UTT people had to go and come back the next day to do baseline bloods and they would not come back again. But now with UTT people start treatment and take bloods on the day they are diagnosed.”* ***(DLHC005_EN_F)***

*“Getting to sit clients in one session and go through everything is better than breaking into several sessions.”* ***(UCSC004_EN_F)***

#### Human resources

In some of the facilities, the implementation of UTT is reported to have improved the skills of HCPs through the trainings provided to them. Also, HCPs reported that more manpower have been hired in some facilities to help ease the workload.

*“Personnel has been hired. Less money spent on minor ailments.” ****(PMSA1_CL_F)***

*“Yes, this is because us as health care providers were given training on HIV testing and treatment.”* ***(KEMG005_EN_F)***

### Perception of UTT on the organization of care in the health system

Participants expressed different views about the effect of UTT on care organization in the health system. They mostly highlighted how the implementation has assisted in decreasing the burden on the health system and helped to attain the NDoH goals of having more individuals linked to care and the provision of immediate access to treatment, to reduce the incidence of HIV infection. These were some of the themes which emerged:

#### Increased number of patients initiated

There was a consensus among HCPs that the UTT strategy has led to an improvement in the number of PLHIV who initiated care and are started on treatment.

*“It [UTT] has increased the number of people getting tested and getting medication soon after they are tested positive.”* ***(ESVC007_CHW_F)***

*“It [UTT] has increased the stats numbers for people initiated on medication.”* ***(ECHC014_EN_F)***

#### Decreased burden on the system

Participants claimed that because of the implementation of the UTT strategy, hospitals no longer experience high admissions and high rates of lost to follow-up of PLHIV due to fewer people getting sick, and the absence of the prescribed pre-antiretroviral therapy services, such as counselling, and clinical staging.

*“[UTT] reduced the number of admissions because clients do not get sick.” ****(KEMP007_EN_F)***

*“[UTT] reduced the number of lost to follow-up because they don’t do pre-ART anymore.”* ***(DIGC011_EN_F)***

*“Many people start treatment before they would get sick, therefore less people are admitted to the hospital for HIV.”* ***(UECG007_EN_F)***

#### Reaching NDoH targets

The main purpose of implementing the UTT strategy was to reduce the incidence of HIV infection through increased HIV testing and provision of immediate access to treatment to PLHIV. Achieving this require the attainment of certain targets. Participants asserted that UTT has increased the number of PLHIV retained in care and are virally suppressed.

*“It [UTT] has increased the numbers of people who are on treatment for HIV which has improved the 90-90-90 programme.” ****(ECHC007_HM_F)***

*“It [UTT] has helped us know the number of people tested positive, initiated, with suppressed viral loads and to reach the 90-90-90.”* ***(ESVC006_LO_F)***

*“It [UTT] has increased the number of people getting tested and getting medication soon after they are tested positive.”* ***(ESVC007_CHW_F)***

#### Financial aspects and stakeholder involvement

Other concerning challenges highlighted by the participants include the need for more medical stocks (ARVs) as the number of patients being initiated on ART increases. In some facilities, HCPs voiced their concern about ARV stock-outs and the increased financial burden that comes with purchasing more ARVs. This is further linked to the lack of involvement of stakeholders (i.e., health care facility managers) in the decisions to implement UTT in under-resourced facilities. Unhappy feelings were expressed by most HCPs on the processes followed to implement UTT. They mentioned not being part of the decision-making team, and highlighted gaps in the UTT implementation processes such as the absence of piloting UTT and the provision of training on some of the processes that were adopted. These were their views:

*“The drugs [ARVs] are running out.” ****(DIGC006EN_F)***

*“The money spent on ART is increasing.”* ***(DIGC010_EN_F)***

*“I feel that we were not involved as people on the ground level; they [policy makers] didn’t consult with us when they were deciding on it [UTT]. So, the only people who were involved were people from the national level and we were not consulted before its implementation as well but were instructed to implement it.”* ***(ESVC001_CM_F)***

## Discussion

South Africa has invested so much in upscaling the UTT strategy with the hopes of improving linkage to and retention in care among PLHIV [16–19]. This study, which aimed to explore HCPs’ perspectives on the implementation of UTT, is one of the very few conducted amongst South African healthcare providers to understand their views and perceptions about the implementation of UTT. Findings from this study highlight the perceived influence of implementing UTT on service users, on healthcare providers, and on the health system in the uThukela DM in KwaZulu-Natal province, South Africa. Our study reveals a general acceptance by healthcare providers of the implementation of the UTT strategy, which came with the understanding that HIV-positive clients will have a better chance of living longer if they are immediately started on treatment. Clinical benefits were also seen as a positive outcome of the implementation of UTT as the UNAIDS 90-90-90 targets were met. In some facilities, enrolled nurses and clinic managers commented on the advantage of implementing UTT as it has improved the clinic flow and operations. These positive views on the implementation of UTT were similarly found in other studies [23, 27]. The acceptance from HCPs who are in the frontline and advocates of the new UTT policy guidelines gives hope that South Africa is on the right direction towards improved rates of HIV testing, linkage to care, and viral load suppression.

Furthermore, HCPs highlighted the positive influences UTT has on the quality of life of patients. They also mentioned that UTT reduced congestion and waiting times at clinics and therefore making clinic flows better for patient satisfaction. Similar findings were reported in other studies where HCPs emphasized patients’ health benefits resulting from the implementation of UTT [27, 43, 44].

The main disadvantage highlighted by HCPs was that UTT might cause an increase in patient defaulters, as some patients may not be ready to immediately initiate treatment [45]. HCPs reported that the increased volume of defaulters has led to a surge in their workload since the implementation of UTT. The challenges about increased workload have been reported in other studies as well [24, 46, 47], and may undermine the quality of care provided by healthcare workers. Improved counselling strategies are needed in these facilities to reduce the number of defaulters caused by overwhelmed patients [26]. One of the crucial aspects of providing quality health care is proper training of HCPs [48], and this could be undermined by the lack of skills and training, and staff shortages. Health system strengthening such as providing more capacity and capability might decrease the already over-burdened system, and thus improve the delivery of the comprehensive UTT services to PLHIV [49]. This study show that training provided to HCPs on the implementation of UTT were insufficient. These issues have been highlighted in other studies [25–29, 47]. Findings from Plazy and colleagues [26] favoured the move from the use of few experienced nurses to an integrated model of care, with a workforce trained in the Nurse Initiated Management of Antiretroviral Therapy (NIMART) [50], who have counselling skills. Other studies support the provision of differentiated service delivery models that have the potential to increase the retention in care rates and adherence to medication among people living with HIV [39, 40].

Another disadvantage of the UTT strategy reported by HCPs includes poor consultation from the National Department of Health on the implementation of the UTT. This may have led to the gaps mentioned by some facility managers, such as insufficient training for HCPs [18] as some of their responses were not in line with the UTT guidelines. To strengthen health system and provide effective UTT services, there is a need to explore better mechanisms to ensure that adequate information is available to HCPs, and that they are provided with the necessary training to offer comprehensive UTT services [27]. The crucial importance of retraining HCPs with new policies can never be over stressed if there must be adequate health workforce to deliver UTT services [18, 26].

This study shows that UTT has led to increased number of patients being tested and immediately initiated on treatment. It is concerning to note that this increase translates to more required medical stocks and that some facilities experience ARV stock-outs and increased financial burden that comes with purchasing more ARVs. Shortages of ARVs can be a barrier in effective implementation of UTT as it means that patients may not be immediately initiated on treatment, and this could have a negative impact on the attainment of the 95-95-95 targets. Shortages of treatment has been reported in at least one out of three facilities in South Africa [18, 31, 32, 51]. For the success of the UTT policy, it is suggested that the NDoH engage fully with stakeholders to ensure that under-resourced facilities are provided with the necessary health equipment and training to enable HCPs offer comprehensive UTT services.

### Study Limitations

A limitation of this study is that most interviews were conducted in clinics, which might have caused discomfort and led to socially desirable responses. However, interviewers were well trained in managing these issues. Results from this study may not be generalizable as the study included a small sample from a single district in KZN province, which may be different from the other eight provinces in South Africa.

## Conclusion

Our results highlight important gaps in the implementation of UTT in a high HIV burden district in South Africa. There is a need for policy makers to engage with facility managers around elements such as infrastructure, human resources, and financial capacity which are crucial in providing a comprehensive UTT programme. For a country such as South Africa, health system level interventions like UTT would need to also focus on clinic readiness in terms of providing patients with the necessary and effective health services such as proper counselling care and psychological assistance to manage their HIV status. This includes proper training for the counsellors to be able to provide appropriate counselling care in the management of patient readiness for the lifelong ART journey. Other health system interventions such as ensuring availability of medicines based on the expected demand, and adequate human resources for the expected increase in workload, could reduce strain on HCPs. This would improve and increase the number of patients who link to care and remain in care, and thus meet the 95-95-95 targets.

More studies are needed on the acceptability of UTT by health care workers in South Africa. Furthermore, for the success of the UTT policy, we recommend that the NDoH engage fully with stakeholders including HCPs to ensure that they are provided with the necessary training to offer comprehensive UTT services. The importance of retraining HCPs with new policies can never be over stressed if there must be adequate health workforce to deliver UTT services.

## Data Availability

The datasets generated and/or analysed during the current study are available from the corresponding author on reasonable request.
